# Clinical and immunological effects of Rituximab in patients with lupus nephritis refractory to conventional therapy: a pilot study

**DOI:** 10.1186/ar1954

**Published:** 2006-05-05

**Authors:** Mónica Vigna-Perez, Berenice Hernández-Castro, Octavio Paredes-Saharopulos, Diana Portales-Pérez, Lourdes Baranda, Carlos Abud-Mendoza, Roberto González-Amaro

**Affiliations:** 1 Departamento de Inmunología, Facultad de Medicina, UASLP, San Luis Potosí, SLP, México; 2 Laboratorio de Inmunología Celular y Molecular, Facultad de Ciencias Químicas, UASLP, San Luis Potosí, SLP, México; 3 Unidad Regional de Reumatología y Osteoporosis, Hospital Central Dr Ignacio Morones Prieto, San Luis Potosí, SLP, México

## Abstract

We studied the clinical and immunological effects of Rituximab (anti-CD20) therapy in patients with lupus nephritis. In an open clinical trial, 22 patients with active systemic lupus erythematosis and renal involvement (mainly class III and IV according to the WHO classification) that was refractory to conventional therapy were studied. In all these patients, Rituximab (0.5 to 1.0 g at days 1 and 15) was added to the immunosuppressive therapy and its therapeutic effect was evaluated. In addition, the levels and function of regulatory T lymphocytes and the apoptosis of immune cells were assessed. We found a significant reduction in disease activity (*p *< 0.05, MEX-SLEDAI index), and proteinuria (*p *< 0.05) at days 60 and 90 of Rituximab therapy. Although most patients showed improvement in creatinine clearance and erythrocyturia, no significant changes in these parameters were detected. In most patients (20/22), B cell depletion was observed, but no clear-cut effect of Rituximab on complement levels or auto-antibody titers was detected (*p *> 0.05 in all cases). One patient died at day 70 with invasive histoplasmosis. No important adverse effects of Rituximab therapy were registered in other patients. A significant enhancement in the levels of different CD4+ regulatory cells (T_REG_, Th3, Tr1), but not CD8+ Ts lymphocytes, was observed at day 30. This increase was sustained for T_REG _cells at day 90, and accompanied by an improvement in their regulatory function. In addition, we observed an unexpected increase in the apoptosis of T cells at day 30. Interestingly, the enhancement in the suppressive function of T_REG _cells was not observed in the two patients that showed the poorest clinical response to Rituximab. We conclude that the data obtained in this open clinical trial suggest that Rituximab is a promising candidate for randomized controlled trials in patients with lupus nephritis refractory to the conventional immunosuppressive therapy. The effects of Rituximab on regulatory cells and apoptosis of T lymphocytes are interesting and its possible role in the putative effect of this biological agent in systemic lupus erythematosis deserves additional studies.

## Introduction

Systemic lupus erythematosus (SLE) is an autoimmune disease of unknown etiology that affects multiple tissues and organs [1]. The main immunological alterations observed in these patients are the loss of tolerance to self-antigens with polyclonal activation of B lymphocytes, production of different auto-antibodies, and an altered function of T cells [2-4]. In addition, several abnormalities of B lymphocytes, including an increased Ca^2+ ^influx, and a decreased fraction of naïve B cells with enhanced levels of circulating plasmablasts have been described [5,6]. Furthermore, B lymphocytes from SLE patients show an increased synthesis of certain cytokines (for example, IL-10), and have an important role as antigen presenting cells, inducing the activation of CD4+ auto-reactive T cells, which in turn allow the activation and differentiation of B cells, and the production of high affinity auto-antibodies [5,7]. Thus, it is very likely that the hyperactivity of B cells and auto-antibody production in SLE depend on the interactions of self antigens with B cell surface immunoglobulin receptors, and the cognate interaction of these cells with helper T lymphocytes. Several subsets of CD4+ cells with immuno-regulatory activity have been described, including T regulatory (T_REG_) lymphocytes, and type-1 T regulatory (Tr1) cells [8,9]. T_REG _lymphocytes are characterized by the expression of CD4 and by high levels of the α chain of IL-2 receptor (CD25) as well as by their lack of responsiveness to antigenic stimulation [8,10]. These CD4+CD25^bright ^CTLA-4+ cells arise from thymus as natural regulatory cells and exert their activity by cell-to-cell contact as well as by inducing the differentiation of CD4+CD25- lymphocytes into regulatory cells [6,10,11]. On the other hand, Tr1 lymphocytes express CD4 and synthesize IL-10 [9]. These regulatory cells are antigen specific lymphocytes derived from conventional CD4+CD25- naive precursors upon exposure to antigen and IL-10 [9,12]. Additional regulatory cells have been described, including the CD8+CD28- suppressor (Ts) lymphocytes, and the CD4+ Th3 cells, which are characterized by the synthesis of transforming growth factor (TGF)-β [13,14].

In recent years, different groups have explored the role of regulatory T cells in the pathogenesis of human autoimmune diseases, including multiple sclerosis, and rheumatoid arthritis [15,16]. In addition, the possible effect of biological therapy with anti-tumor necrosis factor (TNF)-α agents in rheumatoid arthritis patients on the levels and function of T_REG _cells has been recently explored by our and another group [17,18]. In the case of SLE, it has been reported that CD8+ suppressor cells have a defective function [19]. However, in the case of CD4+CD25+ Treg cells, there are only quantitative studies in patients with SLE, and no functional studies have been performed [20,21].

B cell depletion therapy has been recently assayed in patients with different autoimmune diseases, including SLE [22-24]. In this regard, it has been found that patients with SLE that receive the mouse/human chimeric anti-CD20 monoclonal antibody (mAb) Rituximab show clinical improvement and diminution of the abnormalities found in B lymphocytes [25-27]. Rituximab specifically binds to the B cell-specific antigen CD20, a cell surface protein believed to function in B cell cycle initiation and differentiation [28]. CD20 protein is expressed on immature and mature B lymphocytes, but not in early B cells precursors or plasma cells [29]. Rituximab is a very effective B cell depleting agent *in vivo*, inducing the lysis of these cells mediated by complement and by Fc receptor-bearing cytotoxic cells as well as by inducing their programmed cell death [30,31]. This B cell depleting agent does not have an important effect on serum immunoglobulin levels, and SLE patients that receive it show a variable diminution in auto-antibody titers [25-27]. It is feasible, therefore, that Rituximab exerts additional effects on the immune system that account for its effect on disease activity in SLE. In this regard, it has been described that Rituximab therapy is associated with a diminution of expression of CD40L (CD154) and other activation markers of T lymphocytes [32].

In this work, we have performed an open clinical trial with Rituximab in SLE patients, and evaluated their clinical response as well as its effect on the levels and function of regulatory T lymphocytes and the apoptosis of immune cells.

## Materials and methods

### Patients

Twenty-two patients (19 females and 3 males) with a diagnosis of SLE according to the criteria of the American College of Rheumatology were studied. The main clinical data of these patients are given in Table 1. All patients had active disease with renal involvement, which was classified according to the World Health Organization [33]. In most of them, nephropathy was refractory to the administration of conventional immunosuppressive drugs, and two patients had a disease relapse with massive proteinuria despite this type of therapy.

Rituximab was added to the immunosuppressive therapy at a dose of 0.5 to 1.0 g on days 1 and 15. No changes in the therapy with immunosuppressive drugs, including doses of glucocorticoids, were made during the study. Six patients were not receiving glucocorticoids when Rituximab administration was started.

The clinical response to Rituximab was evaluated by routine laboratory tests, and clinical examination at days 30, 60 and 90. Complete renal response was defined as normal serum creatinine, inactive urine sediment, and urinary proteinuria <500 mg/24 h. Partial remission was defined as >40% improvement in the renal parameters that were abnormal at the onset of the study. Immune effects of Rituximab therapy were evaluated at day 30, and 90. The appearance of important adverse effects, defined as a severe infectious process or significant clinical manifestations associated with the administration of intravenous biological agents was registered. In all cases, informed consent was obtained, and the local University ethics committee approved this study.

### Blood samples and cell isolation

Peripheral blood samples were obtained from all patients before (day 0) and at 30 and 90 days after Rituximab therapy. Peripheral blood mononuclear cells (PBMNCs) were isolated by Ficoll-Hypaque density gradient centrifugation and cell viability (trypan blue dye exclusion) was always greater than 95%. CD4+ lymphocytes were purified using a MACS LS separation column (Miltenyi Biotec, Bergisch Gladbach, Germany). In brief, PBMNCs were incubated with a biotinylated antibody cocktail for 15 minutes at 4°C, washed, incubated with anti-Biotin Micro Beads (Miltenyi) for 20 minutes at 4°C and washed. Then, CD4+ lymphocytes were negatively selected using a MACS LS separation column (Miltenyi). For isolation of CD4+CD25+ cells, CD4+ lymphocytes were incubated with anti-CD25 microbeads for 15 minutes at 4°C, followed by positive selection using an additional separation column. CD4+CD25- lymphocytes were also recovered. Cell purity was always greater than 90%, as assessed by flow cytometry analysis.

### Quantification of regulatory T cells

PBMNCs were double immunostained for CD4 and CD25 or CD4 and CTLA-4 using specific fluorescein isothiocyanate (FITC)- or phycoerythrin (PE)-labeled mAb (BD Pharmingen, San Diego, CA, USA), and then analyzed with a FACSCalibur flow cytometer using the Cell Quest software (Becton Dickinson, San Jose, CA, USA). For the detection of CTLA-4, cells were previously fixed with p-formaldehyde, and permeabilized with saponin. Results were expressed as the percent of CD4+CD25^bright ^and CD4+CTLA-4+ lymphocytes. As suggested by Cao *et al*. [34], CD4+CD25^bright ^cells were defined as those CD4+ lymphocytes showing higher expression of CD25 than autologous CD8+ activated *in vitro *with phytohemagglutinin (PHA).

The percent of CD4+ lymphocytes synthesizing IL-10 was determined using the appropriate commercial kit (Miltenyi), following the instructions of the manufacturer. In this assay, cells were first incubated with a catch reagent (a bi-specific dimeric antibody) that binds to a cell surface antigen of leukocytes and is also able to react with the IL-10 secreted by the cell. Then, cells were incubated for 60 minutes at 37°C, washed and labeled with FITC-, and PE-tagged anti-CD4 and anti-IL-10 mAbs. Finally, double positive cells were detected with a FACSCalibur flow cytometer, and results were expressed as the percent of Tr1 lymphocytes. In additional experiments, CD4+ cells with membrane-bound TGF-β were detected by staining PBMNCs with anti-CD4, and anti-TGF-β labeled mAbs and flow cytometry analysis. Finally, Ts lymphocytes (CD8+CD28) were also detected by two-color flow cytometry analysis using specific mAbs.

### Detection of apoptosis

Fresh isolated PBMNCs were fixed and stained by the TUNEL technique using the Apo-Direct kit (BD Pharmingen). After the fluorescent dUTP nick end labeling, cells were analyzed by flow cytometry. In additional experiments, PBMNCs from the same sample were stained with annexin V labeled with FITC and propidium iodide and analyzed in a FACSCalibur flow cytometer. In addition, annexin V staining was combined with mAbs specific for CD3 or CD19. Results were expressed as the percent of positive apoptotic cells.

### Cell proliferation assays

Non-regulatory CD4+CD25- T cells (1 × 10^5^) were mixed or not with CD4+CD25+ regulatory T cells (1 × 10^4^) in the presence of PHA (5.0 μg/ml) and cultured for 72 hours in complete culture medium in 96 well plates. ^3^H-methyl-thymidine (1.0 μCi/well, specific activity 5.0 Ci/mM; New England Nuclear, Boston, MA, USA) was added for the last 12 hours of culture, and at the end of incubation cells were harvested and proliferation was determined using a liquid scintillation counter. All these experiments were run in triplicate and the results were expressed as the percent of cell proliferation, according to the following formula: % cell proliferation = (cpm of CD4+CD25- plus CD4+CD25+ cells/cpm of CD4+CD25- cells alone) × 100.

### Statistical analysis

Data were compared with the Sigma STAT software (SPSS Inc., Chicago, IL, USA) using T paired and correlation (rho Spearman's coefficient) tests, with a level of significance of *p *< 0.05.

## Results

**Figure 1:**
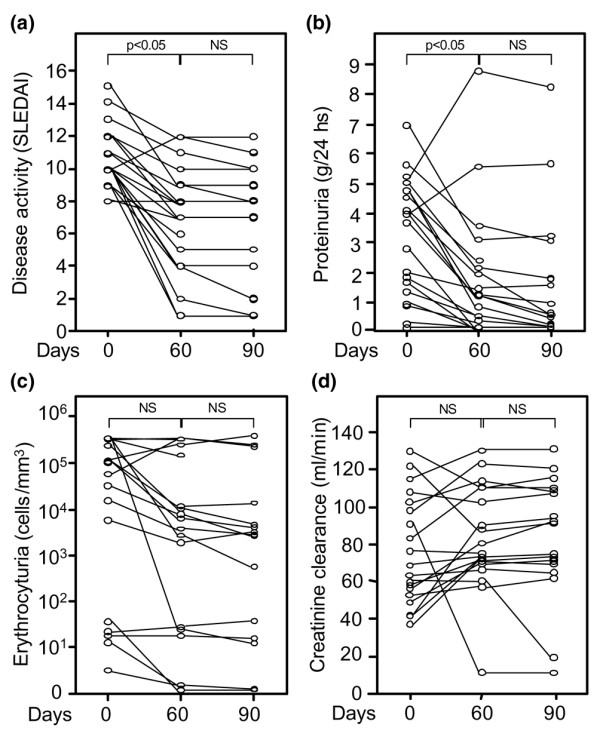
Effect of Rituximab therapy on different laboratory and clinical parameters in patients with systemic lupus erythematosus. Twenty-two systemic lupus erythematosus patients with active disease and renal involvement received Rituximab (0.5 to 1.0 g at days 0 and 15), and the **(a) **disease activity (MEX-SLEDAI index), **(b) **24 hour proteinuria, **(c) **erythrocyturia, and **(d) **creatinine clearance were measured at days 0, 60, and 90.

As previously reported [24-26], we found that Rituximab therapy induced improvements in different clinical parameters of most SLE patients included in this study. Disease activity (MEX-SLEDAI index) significantly diminished in 90% of patients at days 60 and 90 of Rituximab therapy (Figure 1a). In all patients, the dose of glucocorticoids remained unchanged during the study, and in six cases these drugs were not administered (Table 1). Proteinuria levels also significantly diminished, showing an important reduction in most patients (Figure 1b), in some cases as early as day 15, but in other patients at days 60 or 90 of Rituximab therapy (data not shown). However, a substantial enhancement of proteinuria was observed in two patients during Rituximab therapy. Although an improvement in creatinine clearance was observed upon Rituximab administration in 72% of patients studied (Figure 1c), the change in this parameter of renal function was not significant (*p *> 0.05, Wilcoxon sum rank test). Likewise, even though most patients showed reduction in erythrocyturia, this change was not statistically significant (Figure 1d). Based on these results, five patients showed a complete remission of renal disease, whereas seven patients showed a partial renal response. Six additional patients exhibited improvement in one or several renal parameters, but they could not be classified as having a partial or complete remission. Finally, two patients that did not show improvement of any renal function parameter had renal failure at days 60 and 90.

On the other hand, there was a great variability in autoantibody titers (anti-nuclear, anti-dsDNA, anti-phospholipid antibodies), and serum complement levels (CH_50_, C3, C4) upon Rituximab therapy either at days 30, 60, or 90 with no significant changes (*p* > 0.05 in all cases, data not shown). In most patients (20/22), B cell depletion (<5.0 B cells/mm^3^) was induced by Rituximab. Finally, neither serious infectious processes nor important clinical manifestations associated with the administration of the biological agent were observed in 21 out of 22 patients included in the study. However, patient number 18 (Table 1) was admitted to our hospital at day 70 of Rituximab therapy with severe metabolic acidosis associated to diabetes mellitus and pneumonia; 48 hours later, this patient had severe respiratory failure with diffuse pulmonary infiltrates and died. At necropsy, invasive histoplasmosis in the lungs with massive hemorrhage was observed as well as mucormycosis in a coronary artery.

**Figure 2:**
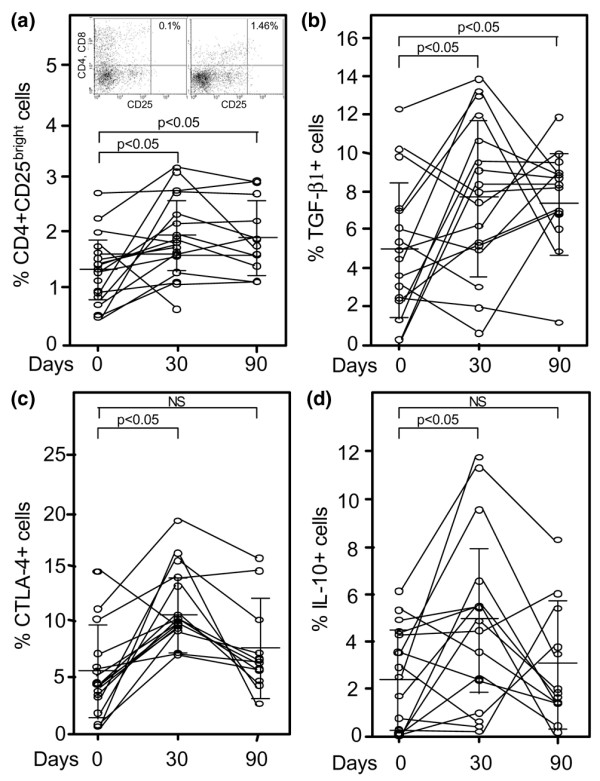
Effect of Rituximab therapy on the levels of peripheral blood regulatory T cells. In 17 systemic lupus erythematosus patients that received Rituximab (0.5 to 1.0 g at days 0 and 15), the levels of **(a) **CD4+CD25^bright ^and **(c) **CD4+CTLA-4+ cells, and CD4+ lymphocytes synthesizing **(b) **TGF-β+ or **(d) **IL-10 was assessed in peripheral blood by flow cytometry before (day 0), and at days 30 and 90 of Rituximab therapy, as described in Materials and methods. The inset in (a) shows how the expression of CD25 by activated CD8+ cells (left histogram) was employed to define CD4+CD25^bright ^cells (right histogram). The arithmetic mean is indicated with a horizontal line, and the standard deviation with a vertical line. In all cases, the percentage of positive cells is referred to the gate of lymphocytes, defined by side and forward scatter characteristics.

Different immunological effects of the Rituximab therapy were assessed in most of the patients (17 out of 22) included in this study. We first determined the levels of T_REG _cells in the peripheral blood of these patients after and before Rituximab therapy. Before starting anti-CD20 administration, we found a variable number of CD4+CD25^bright ^cells in the 17 patients studied, which was significantly increased at days 30 and 90 of Rituximab therapy (*p* < 0.05 compared to day 0, *t* paired test; Figure 2a). Likewise, the levels of other T cells with regulatory phenotype were enhanced upon Rituximab therapy. CD4+TGF-β+ lymphocytes, which very likely correspond to Th3 cells, significantly increased after administration of Rituximab (Figure 2b). A similar phenomenon was observed, at day 30 but not day 90, for CD4+CTLA-4+ and CD4+IL-10+ lymphocytes (Figure 2c,d), a phenotype that corresponds to Tr1 cells. In contrast, no significant changes were observed in the levels of Ts (CD8+CD28-) cells upon Rituximab therapy (*p* > 0.05, data not shown). No significant correlation between the enhancement in the levels of regulatory T cells and the improvement of different clinical parameters was detected (rho Spearman's coefficient; data not shown).

**Figure 3:**
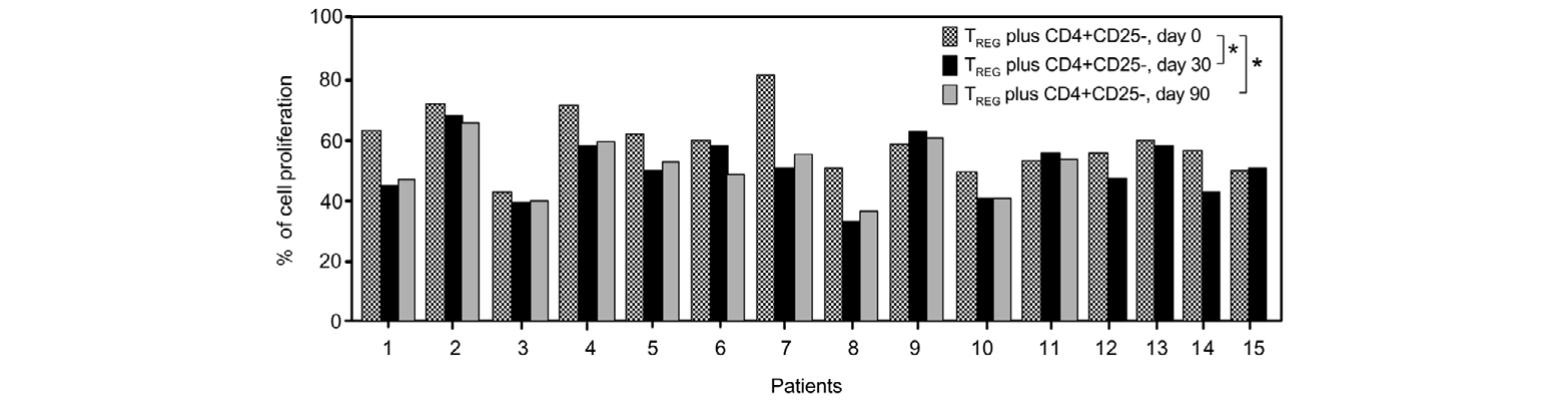
Rituximab effect on the suppressive function of T regulatory (T_REG_) cells in patients with systemic lupus erythematosus. CD4+CD25+ T cells were isolated and then mixed or not with autologous CD4+CD25- lymphocytes in the presence of phytohemagglutinin, and cultured for 72 hours. Then, cell proliferation was determined by ^3^H-methyl-thymidine incorporation. These assays were performed before (day 0), and at days 30 and 90 of Rituximab therapy, as described in Materials and methods. All experiments were run in triplicate, and the results expressed as the percent of cell proliferation (CD4+CD25- lymphocytes cultured without CD4+CD25+ cells = 100%). * *p* < 0.05

We then studied the effect of Rituximab administration on the suppressor activity of T_REG _cells. We found that before starting Rituximab therapy, CD4+CD25+ lymphocytes from patients with SLE showed a variable capability to inhibit the proliferation of autologous CD4+CD25- cells (CD4+CD25+:CD4+CD25- ratio 1:10) stimulated with PHA (Figure 3). At days 30 and 90 of Rituximab therapy, a modest but significant increase in the regulatory function (with a decreased cell proliferation of CD4+CD25- cells) of T_REG _cells was observed (*p *< 0.05 compared to day 0, *t* paired test; Figure 3). Similar results were obtained, but with higher levels of inhibition, when the ratio of regulatory:responder cells was 1:1 (data not shown). As in the case of the levels of regulatory T cells, we did not detect any significant correlation between the enhancement in the suppressive function and the improvement of different clinical parameters (data not shown). However, the two patients (9 and 11) that had the poorest clinical response to Rituximab administration did not show any improvement in the suppressive function of their T_REG _cells (Figure 3). Nevertheless, a modest increase in the levels of CD4+ regulatory T cells was observed in these patients.

**Figure 4:**
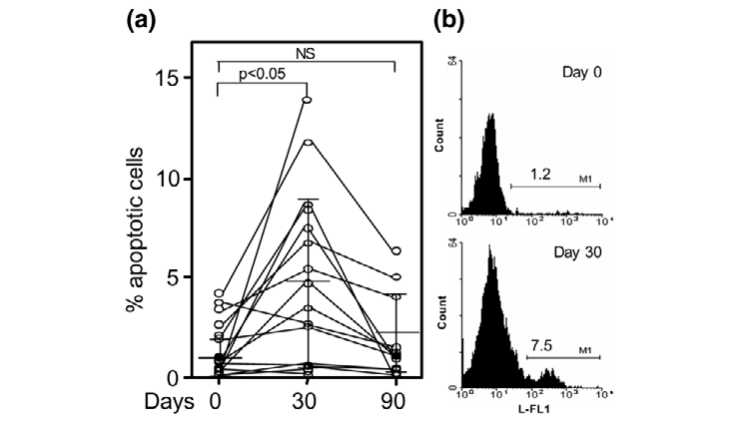
Effect of Rituximab therapy on the apoptosis of peripheral blood mononuclear cells (PBMNCs) in patients with systemic lupus erythematosus. Freshly isolated PBMNCs from patients with systemic lupus erythematosus were fixed, stained using the TUNEL technique, and analyzed by flow cytometry before (day 0), and at days 30 and 90 of Rituximab therapy. Data from all samples studied are shown in **(a)**, and representative histograms of flow cytometry analysis are shown in **(b)**. The arithmetic mean is indicated with a horizontal line and the standard deviation with a vertical line. Numbers in (b) correspond to the percent of positive cells.

**Figure 5:**
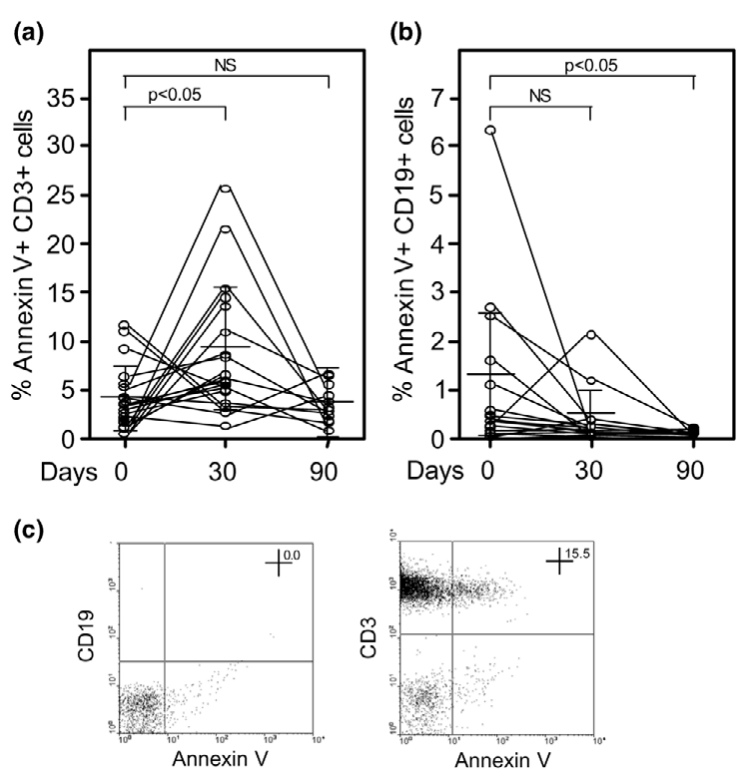
Analysis of apoptosis of subsets of peripheral blood mononuclear cells in patients with systemic lupus erythematosus under Rituximab therapy. Freshly isolated mononuclear cells from systemic lupus erythematosus patients were double labeled with annexin V-FITC plus antibodies specific for **(a) **T cells or **(b) **B cells, and analyzed by flow cytometry, as described in Materials and methods. As indicated, detection of apoptotic cells was performed before (day 0) and after (days 30 and 90) administration of Rituximab. The arithmetic mean is indicated with a horizontal line, and the standard deviation with a vertical line. **(c) **Representative histograms of flow cytometry analysis of CD3+ and CD19+ cells at day 30.

We also analyzed the possible *in vivo *effect of Rituximab on the apoptosis of PBMNCs in the patients included in this study. Interestingly, we found an important and significant increase in the percent of TUNEL+ cells at day 30 of Rituximab therapy (*p *< 0.05, *t *paired test; Figure 4). The presence of apoptotic cells in these samples was confirmed by annexin V labeling and propidium iodide staining (*p *< 0.05, data not shown). Two-color flow cytometry analysis using anti-CD3 and anti-CD19 mAbs plus annexin V-FITC staining showed a significant enhancement in the programmed cell death of T lymphocytes (Figure 5a,c). As expected, very low levels of B lymphocytes, and consequently of apoptotic B cells, were detected in most cases at day 30 of Rituximab administration (Figure 5b,c). When these assays were performed at day 90, we did not further detected a significant enhancement of apoptosis compared to baseline values (Figures 4 and 5).

## Discussion

Different open clinical trials [26,27,35-37] and case reports [38,39] suggest that Rituximab induces a significant reduction in disease activity in patients with severe SLE when it is added to the standard immunosuppressive therapy. However, although it is evident that Rituximab is able to induce an important depletion of B cells in most patients with SLE, the whole consequences of Rituximab administration on the different pathogenic mechanisms of this condition have not been fully elucidated. In this regard, it has been described that therapy with Rituximab has a variable effect on the titers of serum autoantibodies, with a non-significant effect on plasma immunoglobulin levels [25-27,36]. In addition, it has been found that SLE patients receiving this biological agent show a diminished expression of the costimulatory molecules CD40 and CD80 by B cells [40]. Furthermore, Rituximab therapy is associated with a down-regulation of CD40L in T cells as well as a diminution in the expression of activation markers in these lymphocytes [32]. Accordingly, it has been suggested that B lymphocytes participate in the pathogenesis of SLE not only through the synthesis of autoantibodies and that, therefore, Rituximab exerts its therapeutic effect by different mechanisms [24]. To further insight into these putative mechanisms, in this work we decided to evaluate the clinical response to Rituximab as well as to explore its effect on different immunological parameters in patients with SLE.

We selected 22 patients with lupus nephritis that was refractory to conventional therapy with combinations of different immunosuppressive drugs with or without glucocorticoids. Two patients with disease relapse and massive proteinuria were also included. The addition of Rituximab to the ongoing immunosuppressive therapy of these patients significantly diminished disease activity in most of them. These results are in agreement with previous work showing a rapid therapeutic effect of Rituximab in SLE patients with severe disease [26,27,35-40]. In addition, our work suggests that this anti-CD20 mAb could be effective in the absence of glucocorticoid administration (six patients; Table 1), and even as a single immunosuppressive agent (one patient), and as early as at day 15. Furthermore, although we did not observe a significant change in erythrocyturia and creatinine clearance upon Rituximab therapy, our results show that in 77% of cases, this biological agent induced improvement in one or two of these parameters of renal function, even over the short term of this report (60 to 90 days). Therefore, our data further support the therapeutic potential of Rituximab in SLE patients with severe and refractory disease. However, it will be very important to make a further follow-up of our patients (for example, after one year), mainly because of the type of renal disease (III and IV) predominant in them.

It is evident that some patients do not show a satisfactory response to Rituximab. In this regard, it has been described that polymorphisms of the CD64 (FcγRIII) gene have an important role in the B cell depletion effect of Rituximab [41]. Since two patients included in our study showed no B cell depletion (defined as <5.0 B cells/mm^3^) [41] and a poor clinical response to Rituximab, it is very likely that they bear this type of polymorphism. These patients showed a significant increase in proteinuria with an important fall in renal function during the study. The possible presence of anti-chimeric antibodies may also contribute to the lack of effect observed in these cases.

Remarkably, the administration of Rituximab in this study was not accompanied by serious adverse effects in 21 out of 22 patients, despite the long-term depletion of B cells. However, one patient died because of invasive histoplasmosis, massive pulmonary hemorrhage, and mucormycosis. Although this patient was diabetic, and was receiving three immunosuppressive drugs plus high doses of glucocorticoids, it is very feasible that the inhibitory effect of Rituximab on the immune system significantly contributed to the fatal opportunistic infection. Therefore, it would seem necessary to be careful with the administration of Rituximab in patients that are receiving several immunosuppressive drugs. However, it is worth mentioning that in this study, five additional patients were receiving, in combination with Rituximab, three or more immunosuppressive drugs, plus glucocorticoids. As stated above, no adverse effects were observed in these patients.

As in other clinical trials [25-27], we have not found a clear-cut effect of Rituximab therapy on serum autoantibody titers, and even on complement levels. However, we observed significant changes in other immune parameters, including a sustained or transient rise in the proportion of different regulatory T cell subsets in peripheral blood, including T_REG _(CD4+CD25^bright^) lymphocytes, Tr1 (CD4+IL-10+) cells, and Th3 (CD4+TGF-β+) lymphocytes. In most cases, this enhancement was accompanied by an increase in the suppressor function of T_REG _lymphocytes. Although the mechanism responsible for this effect of Rituximab remains to be elucidated, it could be speculated that the decrease of antigen presenting cells induced by Rituximab would induce a decrease in activated T cells, which express, among other cell markers, the ligand of the glucocorticoid-induced TNF receptor (GITRL) [42].

Since it has been described that this ligand is able to inhibit the anergic behavior and suppressive function of T_REG _cells [42,43], it is feasible that a decrease in lymphocytes bearing GITRL favors an increase in the number and function of regulatory cells. In this regard, we have recently found that the inflammatory cell infiltrate of thyroid autoimmune disease contains a high number of GITRL+ lymphocytes, which very likely contribute to inhibit the function of T_REG _cells, (M Marazuela submitted for publication). Another interesting possibility is that the depletion of B cells induced by Rituximab diminishes the synthesis of anti-lymphocyte antibodies, which could be directed against CD4+ regulatory T cells. In any case, it is worth mentioning that our group and others have previously found that other biological agents (anti-TNF-α) are also able to enhance the number and function of T_REG _cells in patients with an autoimmune/inflammatory disease (rheumatoid arthritis) [17,18]. As in the case of Rituximab, the mechanism responsible for this effect of anti-TNF-α agents remains to be elucidated.

It is worth mentioning that the standard assay of suppression of cell proliferation employed by us does not allow us to rule out a possible effect of Rituximab on the non-regulatory cells, increasing their sensitivity to the suppressive signals of T_REG _lymphocytes. In this regard, it has been recently reported that CD4+CD25- cells from MRL/Mp mice show a reduced sensitivity to suppression by autologous Treg lymphocytes. [44], a phenomenon that could be related to defects in intracellular regulatory molecules such as Cbl-b [45]. Thus, it will be interesting to elucidate through future studies whether this effect of Rituximab in SLE patients is mediated by an enhancement of the functional capability of CD4+CD25+ cells or by increasing the sensitivity of effector CD4+CD25- lymphocytes to regulatory signals.

It is well known that Rituximab induces apoptosis of B cells [30,31], and our data show that in most patients included in this study this biological agent was able to cause a sustained depletion of these lymphocytes. In addition, we have found an unexpected induction of apoptosis of T cells upon Rituximab therapy. Since only B cells express CD20, it is evident that this induction of apoptosis is not due to the direct action of this chimeric antibody on T cells. We think that it is feasible that this increase in T cell apoptosis could be due to a diminished availability of antigen presenting cells, a condition that would induce the programmed cell death of activated auto-reactive T cells. In this regard, it has been described that TcR/CD28 engagement induces activation of anti-apoptotic molecules (BAD, Bcl-_XL_) and promotes T cell survival [46,47]. It is also possible that the depletion of B cells drastically reduces the synthesis of certain cytokines (for example, IL-10) that have an important role in the stimulation of expanded T cell subsets (for example, Th2 lymphocytes) in SLE. In this regard, it has been shown that B lymphocytes are the main source of IL-10 in patients with SLE [7]. Although it is evident that these hypotheses require validation with experimental data, we think that our results suggest that Rituximab exerts, through indirect mechanisms, an important effect on both regulatory and effector T cells.

The possible relationship between the immunological effects of Rituximab on T cells observed by us, and its putative therapeutic activity in patients with SLE, remains to be determined. However, it is worth mentioning that the two patients who did not respond to Rituximab in this study did not show significant changes in either the function of regulatory cells or apoptosis of T lymphocytes. In addition, it is evident that the immunological effects observed in this study could contribute to disease remission. Nevertheless, it will be important to make a longer follow-up of our patients, to further corroborate the putative relationship between changes in T cells and the therapeutic effect of Rituximab.

## Conclusion

Our results further support that Rituximab is a promising candidate for randomized controlled trials in patients with SLE, mainly in those with nephritis refractory to conventional immunosuppressive therapy. In addition, we consider that our data regarding the *in vivo *effect of Rituximab on regulatory cells and apoptosis of T lymphocytes are interesting and that its possible role in the putative effect of this biological agent in SLE deserves additional studies.

## Abbreviations

GITRL = glucocorticoid-induced TNF receptor family-related ligand; FITC = fluorescein isothiocyanate; IL = interleukin; mAb = monoclonal antibody; PBMNC = peripheral blood mononuclear cells; SLE = systemic lupus erythematosus; TGF = transforming growth factor; TNF = tumor necrosis factor; Tr1 = type-1 T regulatory; T_REG _= T regulatory.

GITRL = glucocorticoid-induced TNF receptor family-related ligand; FITC = fluorescein isothiocyanate; IL = interleukin; mAb = monoclonal antibody; PBMNC = peripheral blood mononuclear cells; SLE = systemic lupus erythematosus; TGF = transforming growth factor; TNF = tumor necrosis factor; Tr1 = type-1 T regulatory; T_REG _= T regulatory.

## Competing interests

The authors declare that they have no competing interests.

## Authors' contributions

MV-P carried out immunological determinations and drafted the manuscript. BH-C carried out immunological determinations and clinical laboratory tests. OP-S participated in the clinical study of patients and contributed to drafting the manuscript. DP-P contributed to immunological determinations and clinical laboratory tests. LB carried out flow cytometry analysis and participated in the coordination of the study. CA-M participated in the clinical evaluation of patients, conceived the study, and contributed to its design. RG-A conceived the study, contributed to the analysis of clinical and laboratory data, and approved the final manuscript.

## Figures and Tables

**Table 1:** Main clinical data of SLE patients studied

Patient	Age (years)/gender	Disease duration (years)	Main clinical manifestation	Nephritis stage/duration^a^	Disease activity^b^	Previous therapy	Current therapy^c^
1	41/F	15	Ou, Ar, Le, Ly	IV/8	12	GC, Cyc, Aza	GC, Aza
2	24/M	4	Er, Ph, Le	IV/4	10	GC, Aza, Mmf, CsA	GC, Mmf
3	36/F	6	Er, Ph, Ou, Ar, Le, Ly	III/6	14	GC, Aza, CsA	GC, Aza, Mtx
4	19/M	5	Le	IV/4	8	GC, Cyc, Mmf	GC
5	56/F	12	Er, Ph, Ou, Se, Ar	IV/3	15	GC, Cyc, Mmf, Aza	GC, Mmf
6	9/M	1	Er, Ph, Se, Ar	IV/1	12	GC, Mmf, Mtx, Aza, Cy	GC, Aza, Mtx, Mmf
7	26/F	11	Er, Ph, Ou, Se, Ar, Le	IV/4	10	GC, Mtx, Aza	GC, Mtx, Aza
8	31/F	16	Ph, Ou, Se, Ar, CNS	IV/8	10	GC, Cyc, Lfm, Aza, Mtx, Mmf	GC, Aza, Mtx, Mmf, Cyc
9	23/F	3	Ph, Ou, Se, Ar, CNS	IV/3	12	GC, Mmf, Aza, Cyc, Mtx	GC, Mtx, Mmf
10	32/F	6	Er, Ph, Ou, Ar, Ly	IV/6	8	GC, Mtx, Aza, Cyc	Aza
							
11	24/F	2	Er, Ph, Ou, Se	III/2	9	GC, Mtx, Aza	Aza, Mtx, Mmf
12	27/F	13	Er, Ph, Ou, Se, Ly	IV/13	19	GC, Mmf, Cyc, Aza	GC, Aza, Mtx, Mmf
13	43/F	6	Er, Ph, Le	IV/6	9	GC, Mtx, Aza, Pdn	Mtx
14	21/F	8	Er, Ph, Ou, Se, Ly	V/8	9	GC, Mtx, Aza, Cyc, Mmf	None
15	36/F	1	Er, Ph, Ly	V/1	10	GC, Aza	Aza
16	25/F	1	Er, Ou, Se, Ly, CNS	IV/1	11	GC, Aza, Mtx	GC
17	33/F	8	Er, Ph, Ou, Se, Ar, Le	IV/8	10	GC, Aza, Mtx, Mmf, Cq	GC, Aza, Mtx, Mmf
18^d^	28/F	3	Er, Ph, Ou, Se, Le, Ly	IV/3	9	GC, Cyc, Aza, Mmf, Mtx, Cq	GC, Aza, Mtx, Mmf
19	40/F	4	Er, Ph, Ou, Se, Ar	IV/4	10	GC, Aza, Mtx	Aza
20	19/F	1	A, Le, Ly	IV/1	10	GC, Aza, Mtx, Mmf	GC, Aza, Mmf
21	24/F	4	Er, Ph, Le	IV/3	10	GC, Aza, Mtx, Mmf, Cyc	GC, Aza, Mtx
22	22/F	1	Ou, Ar, Le, Se	IV/1	10	GC, Aza, Mtx, Mmf, Cq	GC, Aza, Mtx, Mmf

^a^According to the WHO classification [33]; duration is in years. ^b^According to the MEX-SLEDAI index. ^c^Immunosuppressive therapy to which Rituximab was added. ^d^This patient had invasive histoplasmosis of the lungs with massive hemorrhage and mucormycosis of a coronary artery; she died at day 70 of Rituximab therapy. Ar, arthritis; Aza, azathioprine; Cfm, cyclophosphamide; Cq, chloroquine; CNS, central nervous system disease; Er, erythema; GC, glucocorticoids; Le, leukopenia; Lfm, leflunomide; Ly, severe lymphopenia (lymphocyte count <1,000 cells/mm^3^); Mmf, mycophenolate mofetil; Mtx, methotrexate; N, nephritis; Ou, oral ulcers; Ph, photosensitivity; Se, serositis.
